# Target Activity of *Isaria tenuipes* (Hypocreales: Clavicipitaceae) Fungal Strains against Dengue Vector *Aedes aegypti* (Linn.) and Its Non-Target Activity Against Aquatic Predators

**DOI:** 10.3390/jof6040196

**Published:** 2020-09-29

**Authors:** Sengodan Karthi, Prabhakaran Vasantha-Srinivasan, Raja Ganesan, Venkatachalam Ramasamy, Sengottayan Senthil-Nathan, Hanem F. Khater, Narayanaswamy Radhakrishnan, Kesavan Amala, Tae-Jin Kim, Mohamed A. El-Sheikh, Patcharin Krutmuang

**Affiliations:** 1 Division of Bio Pesticides and Environmental Toxicology, Sri Paramakalyani Centre for Excellence in Environmental Sciences, Manonmaniam Sundaranar University, Alwarkurichi, Tirunelveli 627412, Tamil Nadu, India; karthientomology@gmail.com (S.K.); senthil@msuniv.ac.in (S.S.-N.); 2 Department of Biotechnology, Peter’s Institute of Higher Education and Research, Avadi, Chennai 600054, Tamil Nadu, India; vasanth.bmg@gmail.com (P.V.-S.); amalavenky@gmail.com (K.A.); 3 Department of Biological Science, Pusan National University, Busan 46241, Korea; vraja.ganesan@gmail.com (R.G.); taejinkim77@gmail.com (T.-J.K.); 4 PG and Research Department of Zoology, J.K.K. Nataraja College of Arts and Science, Komarapalayam-638183, Tamil Nadu, India; ramias87@gmail.com; 5 Department of Parasitology, Faculty of Veterinary Medicine, Benha University, Moshtohor, Toukh 13736, Egypt; hanem.salem@fvtm.bu.edu.eg; 6 Department of Biochemistry, St. Peter’s Institute of Higher Education and Research, Avadi, Chennai 600054, Tamil Nadu, India; nrkishnan@gmail.com; 7 Botany and Microbiology Department, College of Science, King Saud University, P.O. Box 2455, Riyadh 11451, Saudi Arabia; melsheikh@ksu.edu.sa; 8 Department of Entomology and Plant Pathology, Faculty of Agriculture, Chiang Mai University, Chiang Mai 50200, Thailand; 9 Innovative Agriculture Research Center, Faculty of Agriculture, Chiang Mai University, Chiang Mai 50200, Thailand

**Keywords:** mycotoxins, entomopathogen, arthropods, CYP450, gut-histology, non-toxicity

## Abstract

The present investigation aimed to determine the fungal toxicity of *Isaria tenuipes* (My-It) against the dengue mosquito vector *Aedes aegypti* L. and its non-target impact against the aquatic predator *Toxorhynchites* *splendens*. Lethal concentrations (LC_50_ and LC_90_) of My-It were observed in 2.27 and 2.93 log ppm dosages, respectively. The sub-lethal dosage (My-It-1 × 10^4^ conidia/mL) displayed a significant oviposition deterrence index and also blocked the fecundity rate of dengue mosquitos in a dose-dependent manner. The level of major detoxifying enzymes, such as carboxylesterase (α-and β-) and SOD, significantly declined in both third and fourth instar larvae at the maximum dosage of My-It 1 × 10^5^ conidia/mL. However, the level of glutathione S-transferase (GST) and cytochrome P-450 (CYP450) declined steadily when the sub-lethal dosage was increased and attained maximum reduction in the enzyme level at the dosage of My-It (1 × 10^5^ conidia/mL). Correspondingly, the gut-histology and photomicrography results made evident that My-It (1 × 10^5^ conidia/mL) heavily damaged the internal gut cells and external physiology of the dengue larvae compared to the control. Moreover, the non-target toxicity against the beneficial predator revealed that My-It at the maximum dosage (1 × 10^20^ conidia/mL) was found to be less toxic with <45% larval toxicity against *Tx.* *splendens*. Thus, the present toxicological research on *Isaria tenuipes* showed that it is target-specific and a potential agent for managing medically threatening arthropods.

## 1. Introduction

Parasites have been a major threat for millions of humans and animals since ancient times, bringing about chronic debilitating and disabling diseases [1]. Mosquitoes (Diptera: Culicidae) are vectors for serious parasites and pathogens, including malaria, filariasis, and important arboviruses, such as dengue virus, yellow fever, chikungunya, West Nile virus, and Zika virus [2,3]. Aedes spp.is a vector transmitting the previously mentioned arboviruses, whose dispersion is wide-reaching [3,4]. Almost 40% of the global population live under hazard of dengue, and yearly, 24,000 deaths are reported. The incidence of dengue viruses has grown intensely around the world in the current scenario. The actual numbers of dengue cases are underreported and many cases are misclassified [4]. Dengue is considered to be an endemic disease prominent in more than a hundred nations, including the Americas, Africa, the Western Pacific, and, more importantly, Southeast Asian countries [5]. Managing this disease is mainly achieved through decreasing mosquito populations [6,7]. Overuse and misuse of synthetic insecticides led to the development of resistance, environmental contamination, toxicity to non-target organisms, and adverse effects on animal and human health; accordingly, there is an urgent need to use eco-smart, bio-rational insecticides including cultural and biological ways which could be integrated into mosquito control strategies [8,9,10,11].

Such alternative approaches through biological ways have been widely recognized for decreasing the selective pressure made by chemical pesticide-resistance against arthropods [11,12]. Among them, microbial toxins can target different life-cycle stages of mosquitoes, and, more importantly, they are harmless to non-targets [13]. Entomopathogenic fungi (EPF) are an active substitute for synthetic chemicals due to their degradability [14,15]. Presently, the mode of activity of fungal strains has revealed several avenues for effective arthropod management [15,16,17]. EPF are a promising agent for arthropod management and the operative fungal strain selection is well established according to its virulence against targeted mosquitoes in the applied settings [18]. 

*Isaria tenuipes* (formerly *Paecilomyces tenuipes*) is a common fungal species that frequently affects major agricultural pests usually belonging to the group lepidopteran [18], and we refer to it as “My-It”. Moreover, it has been found that the *Isaria* fungi hold a diversified blend of chemical derivatives delivered chiefly through non-ribosomal peptide synthetase (NRPS), terpenoid synthetase (TS), polyketide synthase (PKS), etc. [18,19]. All of these active metabolites deliver potential anti-viral, anti-bacterial, and anti-cancer agents [20,21]. There is also other previous research on their biological activity against humans and other beings [18]. Active metabolites derived from *I. tenuipes*, such as cephalosporolides B and F, deliver inhibitory activity against the *Panagrellus redivivus* nematode [22]. Despite such benefits, there is no active research on the biological activity of *I. tenuipes* against mosquito vectors of medical importance. Moreover, their mode of action against mosquito reproduction was also unclear. 

Thus, the present investigation aimed (i) to determine the effective lethal larvicidal dosage of active My-It against the dengue vector *Aedes aegypti;* (ii) to detect the sub-lethal dosage activity of My-It on the reproductive potential in dengue vector; (iii) to detect enzyme regulations in the dengue larvae treated with the sub-lethal dosage of My-It; (iv) to determine the non-target impact of My-It against aquatic mosquito predators sharing the same ecological niche as the dengue vector.

## 2. Methodology

### 2.1. Mosquito Culture

The *Ae. aegypti* larval culture was maintained at the insect toxicology laboratory in St. Peters Institute of Higher Education and Research, Avadi, Chennai from 2019, without disclosure to any prior chemicals, and it was preserved at 26 ± 2 °C at 75–80% relative humidity (RH) under a photoperiod of 14L: 10D. Brewer‘s yeast, ChooStix Biskies-branded dog biscuits, and algae collected from pools in a ratio of 3:2 were fed as a diet to the dengue larvae. The first-generation larvae were used for conducting experiments.

### 2.2. Isaria Tenuipes 

Isolation and maintenance of an *Isaria tenuipes* fungal strain were adapted from our previous research (Vasantha-Srinivasan et al. [15], originally obtained from MTCC (Institute of Microbial Technology (IMT), CSIR, Chandigarh, India). The culture was preserved in a potato dextrose agar (PDA) medium for 14 days at 27 °C. The obtained conidia from the suspension media were prepared using 0.1% Tween 80 diluted using double sterilized distilled water and the conidia were well spun for 20 min to avoid any clumpiness. The number of conidia was counted and we determined their active dosage using fluorescent microscopy (Optika Fluoroscence series B-600TiFL, Italy) at 10× using a Neubauer hemocytometer chamber. Several concentrations were prepared of 1 × 10^2^, 1 × 10^4^, 1 × 10^6^, and 1 × 10^8^ conidia/mL through dilution into double distilled water.

### 2.3. Larvicidal Bioassay

Larvicidal bioassays were adapted based on the methodology of the World Health Organization [4] with slight modifications. The second, third, and fourth instar larvae were transferred into 250 mL sterile plastic containers covering 25 mL of dosage treatments with different discriminate concentration of My-It (1 × 10^2^, 1 × 10^4^, 1 × 10^6^, and 1 × 10^8^ conidia/mL) with the blend of 24 mL de-chlorinated sterile water, along with 1 mL of mycotoxin dosage, and kept at 27 °C. This procedure was replicated three times and one control was kept for each replication, i.e., 20 larvae were used without any chemicals. The mortality of the larvae was documented 24 h post-treatments. The percentage of mortality was deliberate and mortality corrections, wherever required, were analyzed using Abbott’s formula [23]. To determine population growth, water was treated with My-It 1 × 10^3^ and newly emerged larvae were controlled. Each treatment was replicated five times. Percentage of mortality was recorded daily until death.

### 2.4. Oviposition Deterrence Index

Sub-lethal dosages of My-It (1 × 10^1^, 1 × 10^2^, 1 × 10^3^, and 1 × 10^4^ conidia/mL) were mixed thoroughly with 200 mL of rearing food in 300 mL glass jars to obtain the desired dosage for the experiments. The gravid females (20 nos.) were alienated equally between treated and control containers. Throughout the experiments, the female groups were kept isolated for 48 h in mosquito cages (25 × 25 × 30 cm). After counting eggs, the oviposition deterrence index (ODI) was calculated using the formula adapted from Hwang et al. [24].

### 2.5. Fecundity Assay

An identical number of unfed male and female (20 nos. each) dengue mosquitoes were used for fecundity experiments. They were introduced into cages for matting measuring (30 × 30 × 30 cm) at different sub-lethal dosages of My-It (1 × 10^1^, 1 × 10^2^, 1 × 10^3^ and 1 × 10^4^ conidia/mL). After receiving a blood meal, the eggs were collected daily using the small plastic containers containing water as an ovitrap in the cages. Three number of eggs laid in the ovitraps by the female was calculated.

The mean number of eggs laid in the ovitraps by the female (fecundity) was calculated by the number of the eggs laid in the ovitraps divided by the number of females. Adult death during the procedure was also measured.

### 2.6. Enzyme Assays

The third and fourth instars of *Ae. aegypti* previously used in the larval bioassays were thoroughly washed with dechlorinated distilled water then rinsed using sterile tissue paper [25]. The prepared enzyme homogenates were prepared based on our previous research [15] and kept on ice for further enzyme assays. Furthermore, enzyme estimations of carboxylesterase (α and β), SOD, glutathione S-transferase (GST), and CYP450 were analyzed based on the adapted methodology of Thanigaivel et al. [26].

### 2.7. Gut Histological and External Physiological Assay

The My-It-treated (1 × 10^5^ conidia/mL) and control larvae were fixed overnight in Bouin’s solution and then de-hydrated and fixed in blocks using paraffin wax. Microtome (Model: Leica, Germany) larval tissue blocks were fixed on sterile microscopic glass slides and stained using hematoxylin and eosin for examination under a bright field microscope and images were captured under an Optika vision lite microscope (2.0 ML). The captured midgut images of both My-It-treated and control larvae were further compared for toxicological screening.

The photomicrography assay was performed with the previous adapted protocol of Coelho et al. [27] with slight modifications. The My-It-treated and control fourth instar larvae were sequentially stabilized in an ethanol dehydration range from 35–70% for 25 min at 27 °C and fixed on microscopic glass slides. Finally, the sections were observed at 40× magnification under a light microscope (Optika vision lite 2.0 ML).

### 2.8. Non-Target Toxicity Assay

The non-target toxicity assay of My-It against beneficial aquatic organisms was performed according to our previous procedure [15]. The non-target beneficial organism *Toxorhynchites splendens* Wiedemann (Culicidae: Diptera) was tested with lethal dosage of My-It (1 × 10^5^, 1 × 10^10^, 1 × 10^15^ and 1 × 10^20^ conidia/mL) with three replications and each replication contained twenty larvae. Dechlorinated sterile water without the addition of My-It was kept as the negative control. For individual assay, ten replications followed. Finally, the mortality rate was recorded 24 h post-treatment.

### 2.9. Data Analysis

Data from the mortality experiments were analyzed by analysis of variance (ANOVA) of arcsine, logarithmic, and square root transformed percentages, and data were expressed as the means of three replicates. Significant differences between treatment groups were analyzed by Tukey’s multiple range test (significance at *p* < 0.05) using the Minitab^®^17 program. For enzyme activity, Microcal Software (Sigma plot 11) was used. The lethal concentrations required to kill 50% (LC_50_) of larvae in 24 h were calculated by Probit analysis with a dependability interval of 95% using the Minitab^®^17 program.

Mosquito longevity was analyzed using a log-rank χ^2^ test of equality over strata (PROC LIFE Table) along with Formula (1) (Allison [28], 1995) with Minitab^®^ 17 statistical software package (Minitab, State College, PA, USA).

(1) $$Growth\;index=\frac{Percent\;survival\;of\;mosquito}{Duration\;of\;larvae/adult\;larvae/adult\;nymph/adult}$$

## 3. Results

### 3.1. Effect of My-It on Mosquito Survival

**Figure 1:**
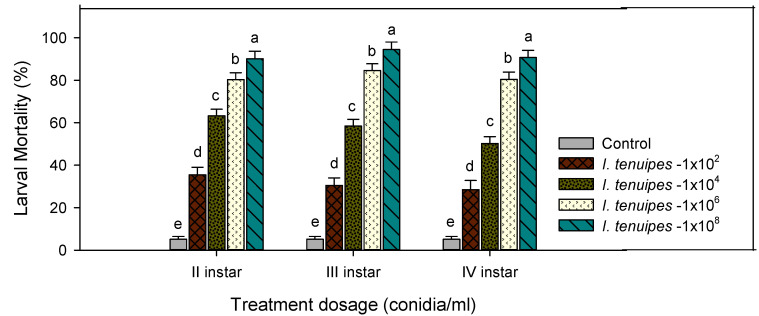
Percentage mortality of second, third and fourth instar larvae of *Ae. aegypti* after treatment with *Isaria tenuipes* conidial spores (My-It). Means (±SE) followed by the same letters above bars indicate no significant difference (*p* ≤ 0.05) using Probit analysis. The different letters (a–e) indicate significant differences between the control and treatments.

The larvicidal activity of My-It with its discriminating dosage (1 × 10^2^, 1 × 10^4^, 1 × 10^6^, and 1 × 10^8^ conidia/mL) against the second instar larvae displayed that the maximum mortality rate of 96% was obtained at the maximum dosage of 1 × 10^8^ conidia/mL and it was more significant than that of the other treatments—1 × 10^6^ (86.32%-F_4,20_ = 16.66, *p* ≤ 0.001), 1 × 10^4^ (63.21%-F_4,20_ = 16.66, *p* ≤ 0.001), 1 × 10^2^ (35.45%-F_4,20_ = 16.66, *p* ≤ 0.001) conidia/mL—and the control (5.140%-F_4,20_ = 16.66, *p* ≤ 0.001) (Figure 1).

**Figure 2:**
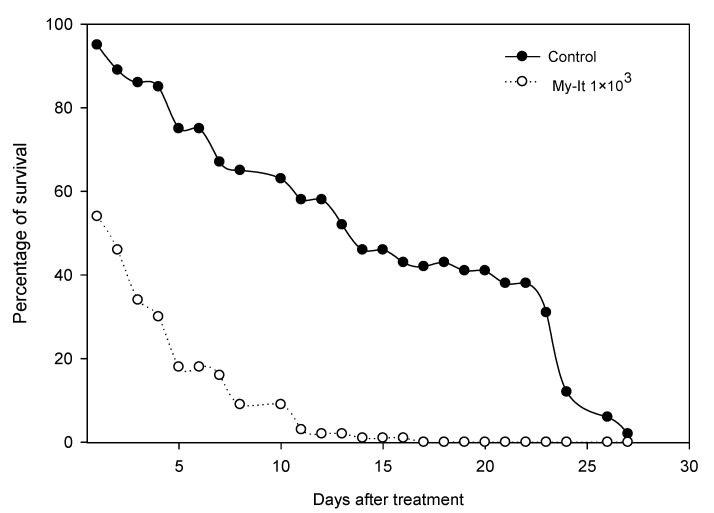
Survival rate of *Ae. aegypti* after treatment with My-It (1 × 10^3^) conidial spores and the control. Survivorship curves differ at the *α* = 0.05 confidence interval according to log-rank statistics.

Similarly, the larval toxicity of My-It against the third instar larvae was also uplifted in a dose dependent manner. At the maximum dosage (1 × 10^8^ conidia/mL), the larval mortality increased significantly to 94.44% (F_4,20_ = 18.22, *p* ≤ 0.001) when compared to those of the other treatments (further total mosquito survival significantly declined when compared with that of the control) (Figure 2).

**Figure 3:**
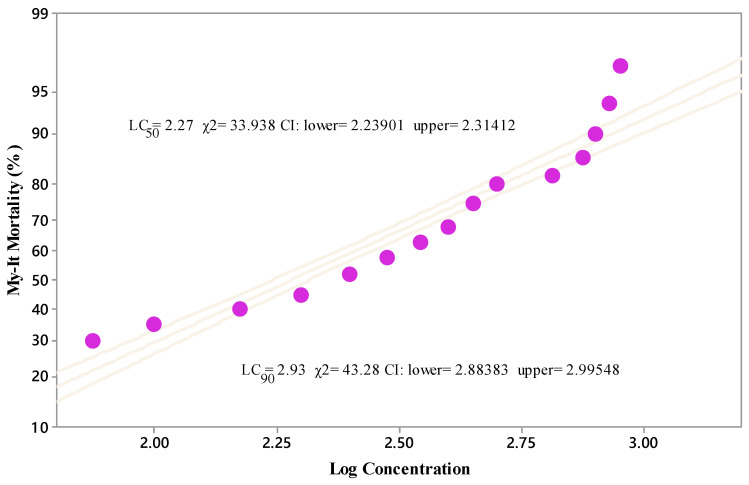
Lethal concentrations (LC_50_ and LC_90_) of My-It conidial spores against fourth instars of *Ae. Aegypti*, obtained using Probit analysis. Dot represents the lethal concentration of *My-It* conidial spores against fourth instar larvae of *Ae. aegypti*.

Likewise, the larvicidal activity of My-It against the fourth instar larvae was increased to 90.67% at the prominent dosage of 1 × 10^8^ conidia/mL and it was significant at 1 × 10^6^ (80.41%-F_4,20_ = 12.33, *p* ≤ 0.001), 1 × 10^4^ (50.18%-F_4,20_ = 12.33, *p* ≤ 0.001), 1 × 10^2^ (28.45%-F_4,20_ = 12.33, *p* ≤ 0.001) conidia/mL and with the control (5.110%-F_4,20_ = 12.33, *p* ≤ 0.001) (Figure 1). The lethal concentrations (LC_50_ and LC_90_) of My-It were 2.27 and 2.93 log ppm, respectively (Figure 3).

### 3.2. Oviposition Deterrence Index 

**Figure 4:**
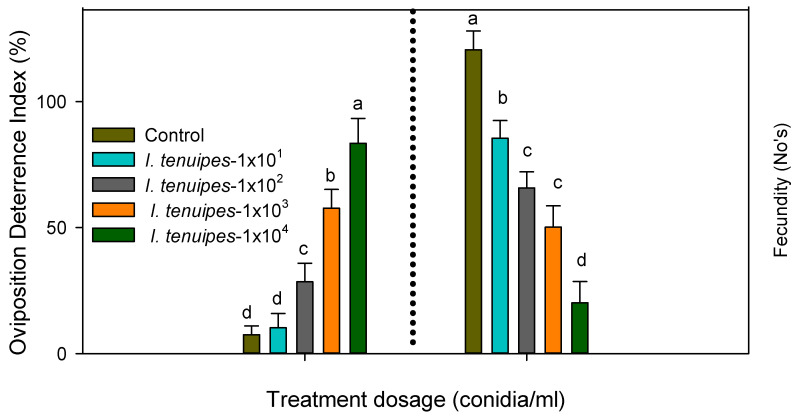
Oviposition deterrent index and fecundity evaluation of My-It against dengue mosquitoes. Means (± SE) followed by the same letters above bars indicated no significant difference (*p* ≤ 0.05) using ANOVA analysis. The different letters (a–d) indicate significant differences between the control and treatments.

The sub-lethal dosage of My-It statistically reduced the oviposition deterrence index of the dengue mosquito at 1 × 10^4^ conidia/mL with maximum deterrence index of 83.4% (F_4,20_ = 25.88, *p* ≤ 0.001) and it is more significant than those of the other treatments—1 × 10^4^ (57.6%-F_4,20_ = 25.88, *p* ≤ 0.001), 1 × 10^3^ (57.6%-F_4,20_ = 25.88, *p* ≤ 0.001), 1 × 10^2^ (28.45%-F_4,20_ = 25.88, *p* ≤ 0.001), 1 × 10^1^ (10.2%-F_4,20_ = 25.88, *p* ≤ 0.001)—as well as the control (7.50%-F*_4,20_* = 25.88, *p* ≤ 0.001) (Figure 4).

### 3.3. Fecundity of My-It

The sub-lethal dosage significantly reduces the mean number of eggs laid by the female dengue mosquito in a dose dependent manner. At the maximum dosage of 1 × 10^4^, My-It showed maximum reduction in fecundity rate (20.1-F_4,20_ = 14.22, *p* ≤ 0.001), followed by 1 × 10^3^ (50.11-F_4,20_ = 14.22, *p* ≤ 0.001), 1 × 10^2^ (66.1-F_4,20_ = 14.22, *p* ≤ 0.001), 1 × 10^1^ (85.45-F_4,20_ = 14.22, *p* ≤ 0.001), and the control (120.45-F_4,20_ = 14.22, *p* ≤ 0.001) mean number of eggs (Figure 4).

### 3.4. Enzyme Inhibition of My-It

**Figure 5:**
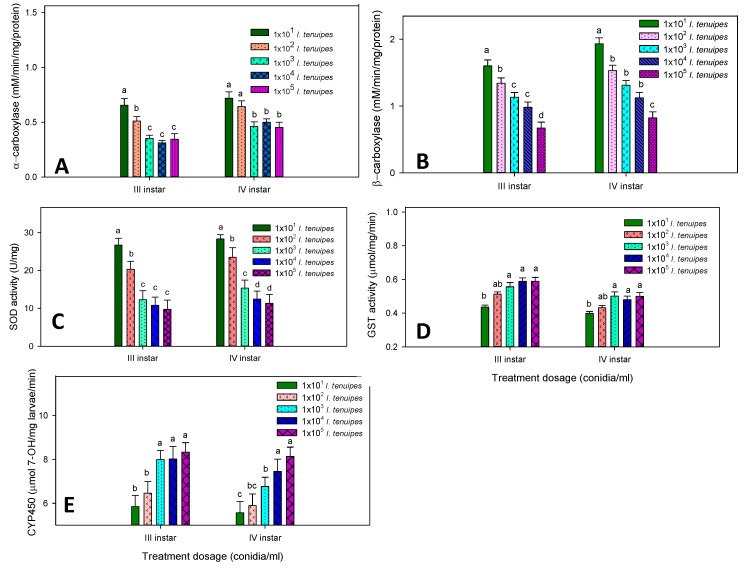
(**A**) α-carboxylestrase; (**B**) β-carboxylestrase; (**C**) SOD; (**D)** GST; (**E**) CYP450 enzyme activity of third and fourth instar larvae of *Ae. aegypti* after treatment with My-It. The data were fitted on a polynomial (regression) model. Letters (a–d) mean (± SE) followed by the same letters above bars indicated no significant difference (*p* ≤ 0.05) using ANOVA analysis.

A sub-lethal dosage of My-It statistically regulates the major enzymes of both third and fourth instars of dengue larvae. The level of α- carboxylesterase was significantly reduced in a concentration dependent manner on both the larval instars. The level of α- carboxylesterase in the third instar reduced at the maximum rate of 0.3451 mg/protein at the maximum dosage of 1 × 10^5^ (F_4,20_ = 18.99, *p* ≤ 0.001), and it was not significant with 1 × 10^4^ (0.312 mg/protein-F_4,20_ = 18.99, *p* ≤ 0.001) and 1 × 10^3^ (0.351 mg/protein-F_4,20_ = 18.99, *p* ≤ 0.001). However, there is no significant difference between 1 × 10^2^ (0.5110 mg/protein-F_4,20_ = 18.99, *p* ≤ 0.001) and 1 × 10^1^ (0.6543 mg/protein-F_4,20_ = 18.99, *p* ≤ 0.001) (Figure 5A). Similar trends were observed in the α- carboxylesterase level in fourth instars with the maximum reduction rate observed in My-It 1 × 10^5^ (0.4514 mg/protein-F_4,20_ = 20.12, *p* ≤ 0.001) which is significant, as is the case with with 1 × 10^2^ and 1 × 10^1^ dosages. However, there was no statistical significance observed with the 1 × 10^4^ and 1 × 10^3^ dosages (F_4,20_ = 20.12, *p* ≤ 0.001) (Figure 5A).

The level of β- carboxylesterase statistically declined at the maximum sub-lethal dosage of My-It of 1 × 10^5^ (0.6700 mg/protein-F_4,20_ = 18.25, *p* ≤ 0.001) and (0.823 mg/protein-F_4,20_ = 16.66, *p* ≤ 0.001) in third and fourth instars, respectively (Figure 5B). However, the level of β- carboxylesterase was 1.600 mg/protein and 1.9320 mg/protein in the third and fourth instars, respectively, at the minimal dosage of My-It (1 × 10^1^) (Figure 5B).

Correspondingly, the level of SOD also declined in a concentration dependent manner with the maximum enzyme reduction observed in the My-It dosage of 1 × 10^5^ conidia/mL with 9.76 U/mg (F_4,20_ = 12.45, *p* ≤ 0.001) and 11.32 U/mg (F_4,20_ = 17.77, *p* ≤ 0.001) in the third and fourth instars, respectively (Figure 5C). However, the level of SOD increased to 26.70 U/mg and 28.32 U/mg in the third and fourth instars, respectively.

The level of glutathione S-transferase uplifted steadily in both third and fourth instar larvae treated with My-It. The level increased to 0.589 mg/min (F_4,20_ = 18.27, *p* ≤ 0.001) and 0.4995 mg/min (F_4,20_ = 12.44, *p* ≤ 0.001) in the third and fourth instar larvae, respectively, treated with My-It 1 × 10^5^ conidia/mL (Figure 5D). However, there is no significant difference between My-It 1 × 10^5^ conidia/mL, My-It 1 × 10^4^ conidia/mL, and My-It 1 × 10^3^ conidia/mL in both of the treated larvae.

The enzyme activity of CYP450 increased in a dose dependent manner with the maximum enzyme rate in My-It 1 × 10^5^ conidia/mL in third (8.3341 µmol 7-OH/mg larvae/min-F_4,20_ = 25.22, *p* ≤ 0.001) and fourth (8.1320 µmol 7-OH/mg larvae/min-F_4,20_ = 18.88, *p* ≤ 0.001) instar larvae, respectively (Figure 5E). In all the treatments, third instar larvae were slightly sensitive in the enzyme regulations of the sub-lethal dosage of My-It (Figure 5).

### 3.5. The Efficacy of of My-It on Gut-Histology 

**Figure 6:**
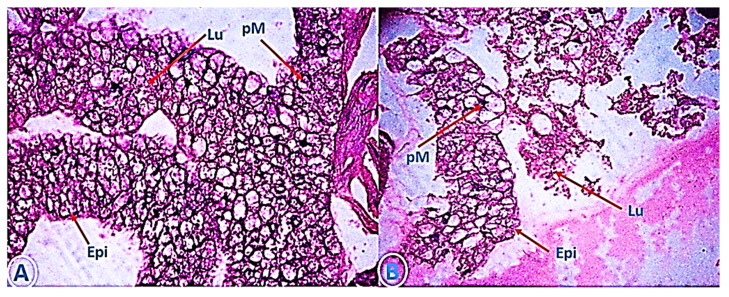
Cross-section through mid-gut of fourth instar *Ae. aegypti* treated with My-It. (**A**), control, compared with (**B**), treated. (Epi) vacuolated gut epithelium; (Lu) gut lumen; (pM) peritrophic membrane.

Treatment with a sublethal dosage of My-It significantly induced adverse effects on the gut though uniformity in the epithelial layer (Epi), gut lumen (Lu), and peritrophic membrane (pM ) were detected in the control larva (Figure 6A), whereas the cellular organelles were severely affected and cranked in the treatment with My-It (1 × 10^5^ conidia/mL) (Figure 6B).

### 3.6. The Efficacy of My-It on the External Physiology of Ae. aegypti Larvae 

**Figure 7:**
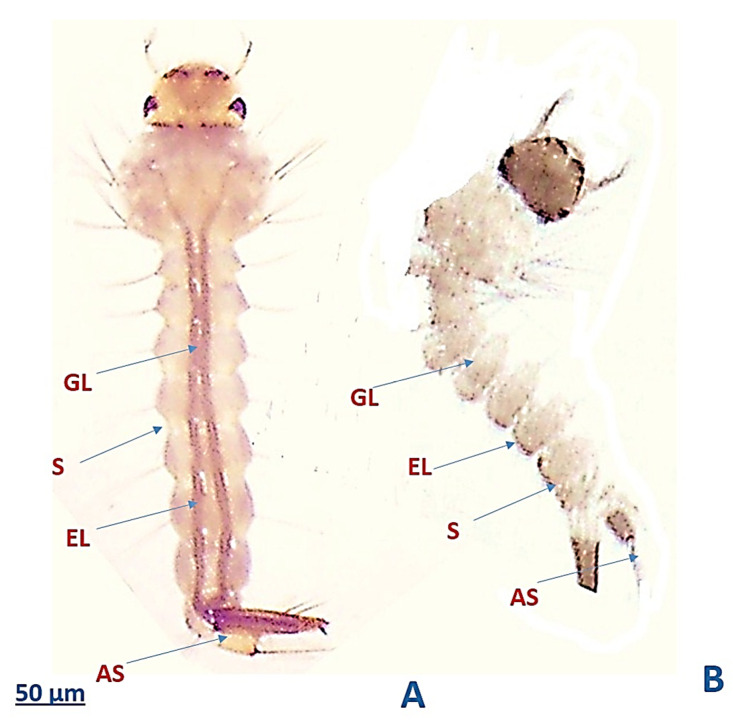
Photo-micrographic analysis of fourth instar larvae of *Ae. aegypti* (**A**) control larvae and (**B**) My-It-treated larvae. (GL)—gut lumen; (S)—segments; (AS)—anal segments; (EL)—epithelial layer.

The external physiological analysis of the fourth instar larvae showed that the sub-lethal dosage of My-It (1 × 10^5^ conidia/mL) drastically affected the gut lumen (GL), segments (S), epithelial layer (EL), and anal segments (AS) (Figure 7B), whereas in the control larvae, the gut cells, including EL, AS, and GL, appeared to be normal (Figure 7A).

### 3.7. Non-Target Toxicity of My-It

**Figure 8:**
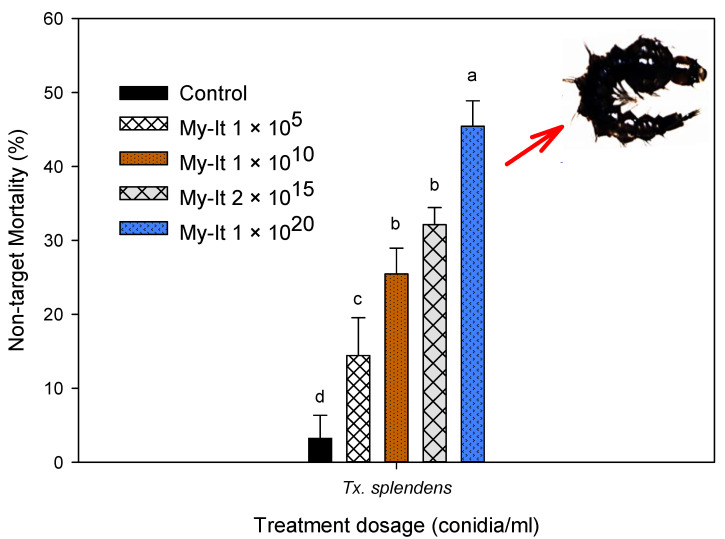
Impact of My-It on the non-target organism *Tx. splendens*. Letters (a–d) mean (± SE) followed by the same letters above bars indicate no significant difference (*p* ≤ 0.05) using Probit analysis.

The non-target toxicity of the aquatic predator *Tx. Splendens*-discriminating dosage of My-It 1 × 10^20^ (3 to 4 fold higher dosages used in larvicidal assay) displayed a lower mortality rate (45.43%-F_4,20_ = 25.66, *p* ≤ 0.001), followed by My-It 1 × 10^15^ (32.14%-F_4,20_ = 25.66, *p* ≤ 0.001), My-It 1 × 10^10^ (25.40%-F_4,20_ = 25.66, *p* ≤ 0.001), My-It 1 × 10^5^ (14.43%-F_4,20_ = 25.66, *p* ≤ 0.001), and the control (3.20%-F_4,20_ = 25.66, *p* ≤ 0.001). There is significant difference between the My-It treatments and the control (Figure 8).

## 4. Discussion

Determining the mosquito resistance pattern against different groups of synthetic chemicals displays a significant role in managing arthropod vectors [29]. Due to up-surging resistance observed in synthetic chemicals, there is an urgent need for novel substitutes to managing blood-sucking pests [30,31]. Novel discovery of natural materials with diversified blends of active ingredients with potential insecticidal properties may provide a suitable remedy for synthetic chemical resistance [32,33,34]. Among different biological insecticides, fungal strains have unique modes of action by penetrating the cuticle and blocking the development of pests [35]. Fungal strains generate a vast range of chemicals with an extensive band of actions against insect pests [36]. Amid fungi, the genus Isaria is an important fungal strain composing >100 different species playing a significant role in conserving biodiversity and that are widely used in agriculture and medical treatment [18,37,38]. *I. tenuipes* is most common species of “*Isaria”* with a wide range of insecticidal activity, especially against agriculture pests belonging to the lepidopteran group [39].

The present study revealed that larval toxicity of My-It displayed a significant mortality rate at the maximum dosage of 1 × 10^8^ conidia/mL, with a larval mortality rate of more than 94% recorded in all the treated larval instars. Similar to our results, mycotoxins derived from *Aspergillus flavus* also displayed prominent mortality rates of more than 90% at the maximum lethal dosage of 2 × 10^8^ conidia/mL [15]. Generally, fungal strains enter the body of a mosquito and create a way to the hemocoel and deliver humoral and cellular immune defensive mechanism straddling by the host of the mosquito species as it tries to overawe the mycotoxin infections [40,41]. Similar to the above statements, My-It delivers acute toxicity to the different instars of dengue mosquito vector.

In general, oviposition represents the vital position in the life cycle of any arthropods, as oviposition inhibition directly signifies the reduction rate in the growth and development of pest populations [42]. Similar to the larvicidal activity, the sub-lethal dosage of My-It significantly affects the reproduction stage of dengue mosquito in dose dependent manner. The sub-lethal dosage of My-It (1 × 10^4^) delivered significant ODI percentage as compared to the control; likewise, fungal strains can significantly inhibit the oviposition of other agriculture pests [36]. Previously, the phyto-pathogenic fungal strains derived from *Botrytis cinerea* blocked the oviposition of a major European insect pest (grapevine moth), *Lobesia botrana* [43]. Likewise, the mean number of eggs laid by the gravid female mosquito (fecundity rate) was also reduced considerably due to the maximum sub-lethal dosage of My-It (1 × 10^4^). The volatile and non-volatile metabolites of fungal strains play a significant role in blocking the fecundity rate of arthropods, especially blood-sucking pests [44]. Similar to the above statement, the major allelochemicals in My-It might cause the blockage of the egg-laying capability of female dengue vectors. Similar to our report, a previous in vitro assay revealed that fungal toxins marginally declined the fecundity rate [45]. Similarly, a previous review by Ondiaka et al. [46] stated that the entomo-toxin derived from *Metarhizium anisoplia* displayed a significant fecundity rate against different insect pests.

Generally, insect resistance against any chemical toxins can primarily be accessed through investigating the level of key biomarker enzymes, including detoxifying and digestive enzymes, such as carboxylesterase, SOD, glutathione S-transferase, and, more importantly, the chief detoxifying enzyme cytochrome P-450 [47]. In the present investigation, the sub-lethal dosage of My-It (1 × 10^5^ conidia/mL) heavily reduced carboxylesterase (both α and β) enzyme regulation ratios in a dose dependent manner. In support of our findings, the sub-lethal dosage of *A. flavus* heavily inhibited the level of both α-β-carboxylesterase and SOD activity [15]. Generally, upregulation of esterase activity will deliver substantial insect resistance against the specific chemicals tested. Resistance developed in esterase-related protein delivered regulatory alterations in the structural genes by modifying the loci of specific genes in insects and also amplified the DNA methylation genes in insect pests [48]. Likewise, Hemingway and Ranson [49] reported that the enzyme families esterase, CYP450s, GST, and SOD are the major four enzymes responsible for pest resistance against the chemical toxins. Backing the above statement, the mycotoxins derived from *I. tenuipes* delivered significant reduction in the carboxylesterase and SOD activity and also delivered a substantial increase in the rate of GST and CYP450 levels.

The gut-histology and physiological alterations results clearly evident that My-It heavily damaged the internal gut cells and external physiology of dengue larvae compared to the control. Similarly, the sub-lethal dosage of *A. flavus* considerably injured the gut epithelial and lumen cells of *Ae. aegypti* larvae [15]. Comparably, a previous review by Rudin and Hecker [50] stated that the pM (peritrophic membrane) stimulates parasite growth in mosquitoes by developing barriers. The above statement was well supported by our present study which shows that the sub-lethal dosage of My-It affected the gut cells of dengue larvae.

It is essential to gage the primary and secondary impact of any forms of pesticides upon non-target species [26,31,51]. *Toxorhynchites* are an excellent predator against the dengue larvae ‘*Aedes*’ and are measured to be not hurtful to their well-beings and well-fixed as they are non-blood feeders and considered to be a good biological predator for reducing the populations of blood-sucking mosquitoes [52]. Since they share the same ecological regions as dengue larvae, it is essential to investigate the non-toxicity screening of same chemicals tested against dengue larvae. The non-target screening of My-It against the giant mosquito (*Tx. splendens*) showed they are less at-risk (maximum 45% mortality rate), even if they are treated with the maximum dosage of My-It (1 × 10^20^) which is the highest dosage used in the larvicidal assays. It is evident that biologically-derived pesticides, especially mycotoxins and their related compounds, were target specific and harmless or less toxic to the beneficial species. Thus, the present toxicological investigation of My-It recommends that it is a highly favorable biological agent in managing medically-challenging arthropods, especially the dengue mosquito, and its non-toxic activity against aquatic predators will add on to its biologically safe insecticides. Further investigation on the active allelochemicals of My-It and its specific mode of action against the dengue mosquito vector’s biological activity needs to be intensely inspected.
